# Exploring Food Insecurity and Nutritional Challenges Among Rickshaw Pullers in Dhaka City, Bangladesh

**DOI:** 10.1002/puh2.70149

**Published:** 2025-10-22

**Authors:** Md. Shahadoth Hossain, Puja Halder, Nafisa Rumman Urbi, Ishra Fairooz Tapti, Wana Marzia, Md. Ruhul Amin

**Affiliations:** ^1^ Institute of Nutrition and Food Science University of Dhaka Dhaka Bangladesh; ^2^ Department of Nutrition and Food Engineering Daffodil International University Dhaka Bangladesh; ^3^ Department of Food & Nutrition Govt. College of Applied Human Science Dhaka Bangladesh

**Keywords:** Bangladesh, diet quality, food insecurity, nutritional status, rickshaw puller

## Abstract

Rickshaw pullers in Dhaka city represent a significant portion of the urban workforce, often facing challenges related to food insecurity (FI) and nutrition. This study aimed to assess the FI status, diet quality, and nutritional status of rickshaw pullers in Dhaka city. A cross‐sectional study was conducted across 10 locations in Dhaka city, enrolling 400 rickshaw pullers through convenience sampling. FI was assessed using the food insecurity experience scale (FIES), and dietary data were collected using the diet quality questionnaire (DQQ). Nutritional status was evaluated through anthropometric measurements. Logistic regression models were employed to identify factors associated with the outcome variables. Approximately 60% of the participants experienced moderate‐to‐severe food insecurity, with 6.8% classified as severely food insecure. The majority (57.3%) reported having debt, and more than half (52.5%) expected financial support to improve their living standards. Participants with higher education (adjusted odds ratio [AOR] = 0.56, *p* = 0.042) and higher household income (AOR = 0.20, *p* < 0.001) had lower odds of experiencing FI. Nearly half (48.8%) of the participants consumed a less diversified diet, with fewer than five food groups. Higher education levels (AOR = 2.74, *p* = 0.001) and a greater number of earning members (AOR = 2.30, *p* = 0.003) were significantly associated with better dietary diversity. About 9% of participants were underweight, whereas 10.4% were overweight. This study highlights a high prevalence of FI, financial strain, and inadequate dietary diversity among participants, with the coexistence of both underweight and overweight individuals. Targeted interventions, including food assistance programs, financial support, nutrition education, and workplace nutrition initiatives, are essential to improve food security and overall nutritional well‐being.

## Graphical abstract

Rickshaw pullers in Dhaka city experience high food insecurity, poor diet quality, and a dual burden of malnutrition. These findings underscore the urgent need for targeted nutrition policies and interventions to improve their health and well‐being.

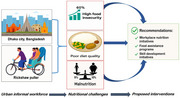

## 1 Introduction

Pulling a rickshaw demands significant mental and physical exertion, yet those who do it often face neglect and harassment from law enforcement, passengers, motorized vehicle workers, and sometimes even rickshaw owners [1]. The vulnerability of rickshaw pullers is exacerbated by the predominant non‐ownership status among those who operate rickshaws, coupled with ongoing challenges and heightened competition within the rickshaw‐pulling community. Moreover, they live a substandard life in Dhaka city, and a majority of them are being deprived of basic facilities as well as their labor rights [2]. Typically, these individuals reside in slums or less developed areas within the city, where living conditions may be characterized by lower infrastructure standards and limited access to essential services [3]. The correlation between income and crucial aspects like food security and diet quality significantly impacts the nutritional well‐being of low‐income individuals [4]. Unfortunately, those with limited financial resources often experience suboptimal levels of food security and diet quality. Consequently, their overall nutritional status is compromised, which directly influences their health and well‐being.

Nutritional status pertains to an individual's health condition, which is determined by their diet and the nutrients obtained through daily food intake, and it serves as a crucial measure of overall health and well‐being [5]. Food security and diet quality are critical determinants of nutritional status, particularly among individuals with lower incomes [6, 7]. Food security exists when all people, at all times, have physical, social, and economic access to sufficient, safe, and nutritious food that meets their dietary needs and food preferences for an active and healthy life [8], whereas diet quality refers to a diversified, balanced, and healthy diet, which provides energy and all essential nutrients for growth and a healthy and active life [9].

Rickshaws serve as prevalent modes of transportation for carrying passengers, luggage, and necessary commodities across diverse urban, suburban, and rural landscapes in Bangladesh, alongside numerous Southeast Asian nations, including India, China, Vietnam, Singapore, and Hong Kong [10]. In Bangladesh, over 5 million individuals rely on rickshaw pulling as a primary source of livelihood [11]. A significant portion of rickshaw pullers in Bangladesh reside in the capital city, Dhaka. Presently, it is estimated that nearly 1.5 million rickshaw pullers, and their families depend on this mode of transportation [12]. The Dhaka North City Corporation (DNCC) has 30,162 registered rickshaws, whereas the Dhaka South City Corporation (DSCC) accounts for 190,217 registered rickshaws [11]. However, a 2019 study conducted by the Bangladesh Institute of Labor Studies (BILS) estimated that the total number of pedal rickshaws in Dhaka exceeds 1.1 million, with only 3%–4% of these rickshaws being officially registered [13]. In Dhaka, around 60% of residents rely on rickshaws for daily commuting. This sector predominantly employs unskilled labor due to its low investment, easy entry, cash‐based income, lack of specialized skills required, widespread availability, and straightforward operational regulations.

Despite their significant presence in the workforce, the lifestyle, dietary habits, and health status of rickshaw pullers in Dhaka and across Bangladesh have largely remained unexplored. Although several studies have been conducted in Bangladesh on various aspects of rickshaw pullers’ lives, significant gaps remain, particularly regarding the challenges they face in their daily routines. One study examined the socioeconomic profile and health conditions of rickshaw pullers, but it was limited to rural settings and involved an insufficient sample size [14]. Two other studies focused on rickshaw pullers in Dhaka city—One addressed only the socioeconomic status of this workforce [15], whereas the other explored their socioeconomic profile, nutritional status, and dietary gaps [10]. However, none of these studies investigated the issue of food insecurity (FI), a critical aspect affecting this substantial labor force. As FI among rickshaw pullers remains unexplored, its impact on diet quality and nutritional status, as well as their coping strategies to mitigate these challenges, remains unknown.

The objective of this study was to investigate the food security status, diet quality, and nutritional status of rickshaw pullers in Dhaka city. These marginalized and exploited groups are not explicitly acknowledged in government policy documents, and very little attention has been paid to humanizing the livelihood of the rickshaw pullers [16]. Therefore, the results of this study may bring their actual situation before society, and appropriate policies may be framed to solve their problems.

## 2 Materials and Methods

### 2.1 Study Design and Participants

This cross‐sectional study was conducted among 400 rickshaw pullers across 10 randomly selected areas in Dhaka city, Bangladesh (Figure S1). The sample size was determined using the standard formula for a cross‐sectional study design [*n* = *Z* ^2^ × *p*(1 − *p*)/*d* ^2^], where estimated proportion, *p* = 50%, margin of error, *d* = 0.05, and *Z* = 1.96 for a 95% confidence interval (CI). Despite an estimated sample size of 384, we successfully enrolled 400 participants. The study participants were selected through convenience sampling.

### 2.2 Data Collection

A structured questionnaire was administered to gather comprehensive data on sociodemographic characteristics, nutritional knowledge, FI, diet quality, anthropometric measurements, and expected support and plans for improving living standards among the study participants. The data collection period spanned from December 2023 to January 2024.

### 2.3 Assessment of Nutritional Knowledge

Nutritional knowledge was assessed using a structured set of 10 questions covering nutrition, healthy eating habits, and health awareness, with each correct response assigned a score of 1. The total scores were then classified into three categories on the basis of Bloom's cutoff criteria: poor (<60%), average (60%–79%), and good (80%–100%) [17].

### 2.4 Assessment of FI

The food insecurity experience scale (FIES) was used to assess FI at the individual level in this study. FIES provides an experience‐based metric for measuring the severity of FI among individuals or households [18]. This scale consists of eight questions that capture varying levels of FI, with the first question addressing mild FI and the final question reflecting severe food insecurity (SFI). Participants answered these questions using binary (yes or no) responses, which were aggregated into raw scores ranging from 0 to 8. On the basis of these scores, individuals were classified into three categories: food secure (FS) with a score of 0, moderate food insecurity (MFI) with scores from 1 to 6, and SFI with scores from 7 to 8. Data from the FIES questionnaire were processed and analyzed using Microsoft Excel and an online platform called Shiny Apps.

### 2.5 Assessment of Diet Quality

The diet quality questionnaire (DQQ) was utilized to evaluate diet quality indicators among the study participants [19]. The DQQ serves as a standardized tool designed to collect data on indicators of nutritional adequacy. It encompasses assessments of various facets, including the minimum dietary diversity for women (MDD‐W) indicator, food group diversity, markers of health protection against noncommunicable diseases (NCDs), and the global dietary recommendation score (GDR) [20]. Food group diversity and prevalence of consumption of food items associated with NCDs protection and NCDs risk were calculated following the guidelines outlined in the DQQ indicator manual [21]. Food group consumption patterns over the week were determined using the food consumption score (FCS) data.

### 2.6 Assessment of Nutritional Status

The weight of the participants was measured using a portable electronic scale (Tanita HA‐650, Japan) with a precision of 0.01 kg, whereas height was assessed using a portable stadiometer, recorded to the nearest centimeter. Body mass index (BMI) was calculated by dividing the weight in kilograms by the square of the height in meters (kg/m^2^). Nutritional status categories were determined on the basis of World Health Organization (WHO) criteria [22]: underweight (BMI < 18.5 kg/m^2^), normal weight (BMI 18.5–24.9 kg/m^2^), overweight (BMI 25–29.9 kg/m^2^), and obese (BMI ≥ 30.0 kg/m^2^).

### 2.7 Explanatory Variables

Age in years (<30, 30–45, ≥46), education level (no formal education, primary, and secondary), household size (small [≤4 members], large [>4 members]), daily working hours (≤8 h, >8 h), earning members (single, two, or more), household monthly income in Bangladeshi taka (BDT) (up to 16,000, 16,001–20,000, 20,001–25,000, >25,000), household monthly food expenditure in BDT (<5000, 5000–8000, 8001–10,000, >10,000), and nutritional knowledge level (poor [<60%], average [60%–79%], and good [≥80%]).

### 2.8 Statistical Analysis

Descriptive analyses were conducted to summarize the data using frequencies and percentages. Two logistic regression models were employed to examine associations within the study. The first model assessed the relationship between FI and sociodemographic variables, whereas the second model analyzed the association between food group diversity and other variables. Variables with *p* values < 0.25 in the bivariate analyses were considered for inclusion in the regression models [23]. Before finalizing the models, the underlying assumptions of logistic regression were assessed (Tables S1 and S2). Multicollinearity among covariates was assessed using the variance inflation factor (VIF), with a threshold of VIF < 2 [24]. Statistical significance was determined at a *p* value threshold of <0.05. Associations were reported as adjusted odds ratios (AORs) with 95% CIs. All analyses were performed using the Statistical Package for the Social Sciences (SPSS), version 26.0.

## 3 Results

Table 1 presents the sociodemographic characteristics of the study participants (*n* = 400). The majority of participants were aged 30–45 years (51.0%). A substantial portion of the population had no formal education (50.5%), and most participants worked more than 8 h daily (63.5%). Additionally, 66.3% of households had a single earning member. Income distribution was fairly even across categories, with an average monthly income of BDT 20,000 (not shown in the table). Regarding food expenditure, 42% households spent between BDT 5000 and 8000. The largest group (45.0%) had an average level of nutritional knowledge.

**TABLE 1:** Sociodemographic characteristics of the study population (*n* = 400)

Variable	Frequency (*n*)	Percentage
**Age (year)**		
<30	67	16.8
30–45	204	51.0
≥46	129	32.3
**Education level**		
No formal education	202	50.5
Primary education	114	28.5
Secondary education	84	21.0
**Household size**		
Small (≤4 members)	203	50.7
Large (>4 members)	197	49.3
**Working hour**		
≤8	146	36.5
>8	254	63.5
**Earning members**		
Single	265	66.3
Two or more	135	33.8
**Household monthly income (BDT)**		
Up to 16,000	105	26.3
16,001–20,000	109	27.3
20,001–25,000	105	26.3
>25,000	81	20.3
**Household food expenditure (BDT)**		
<5000	37	9.3
5000–8000	168	42.0
8001–10,000	119	29.8
>10,000	76	19.0
**Nutritional knowledge level**		
Poor (<60%)	135	33.8
Average (60%–79%)	180	45.0
Good (≥80%)	85	21.2

*Note:* $1 = 121.27 BDT.

Abbreviation: BDT, Bangladeshi taka.

The FI status, coping strategies, nutritional status, and common health conditions among the study participants are presented in Table 2. The results indicate that 59.7% of participants experienced MFI to SFI, with 6.8% classified as severely food insecure. To manage FI, 67.5% of participants reported consuming less preferred and more affordable food options. Regarding nutritional status, 8.8% of participants were underweight, whereas 10.4% were overweight. Fever was the most frequently reported illness (30.7%), followed by other health issues (9.0%), whereas chest pain (1.5%) and heart disease (2.2%) were the least commonly reported conditions.

**TABLE 2:** Food insecurity, coping strategy, nutritional status, and common health problems among the study participants.

Characteristics	Percent
**Food insecurity**	
Moderate to severe	59.7
Severe	6.8
**Reduced coping strategy**	
Relying on less preferred and less expensive food	67.5
Borrowing food or relying on help from others	19.3
Reducing the meal frequency	13.0
Eating less at meal times	23.0
Reducing the quantity of food for adults	10.0
**Nutritional status**	
Underweight (BMI <18.5)	8.8
Normal (BMI 18.5–24.9)	80.8
Overweight (BMI ≥25)	10.4
**Common health problems**
Diarrhea	2.7
Fever	30.7
Abdominal pain	3.5
Headache	3.0
Heart disease	2.2
Chest pain	1.5
Others	9.0

Abbreviation: BMI, body mass index.

**FIGURE 1:**
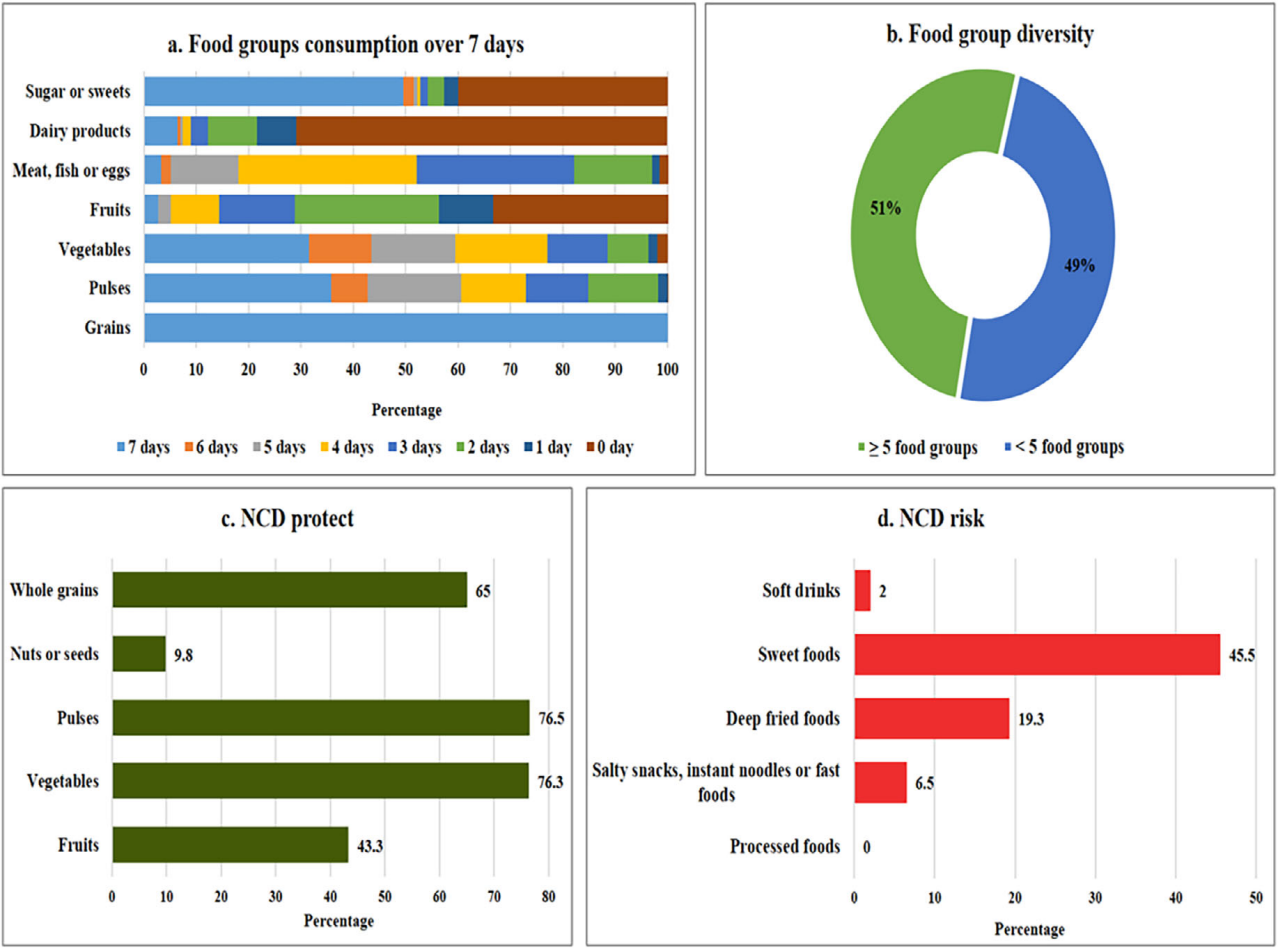
Diet quality indicators among study participants: (a) food groups consumption over 7 days, (b) food group diversity, (c) NCD protect food groups, and (d) NCD risk food groups. NCD, noncommunicable disease.

Figure 1 illustrates the distribution of diet quality indicators among the study participants. The findings show that all of the participants consumed grains daily throughout the week. A considerable portion regularly consumed pulses, vegetables, and sweets, whereas the intake of other food groups was relatively low, particularly dairy products and fruits. Additionally, 51.2% of participants reported consuming a diversified diet with five or more food groups. Most of the participants consumed protective food groups for NCDs, such as vegetables (76.3%), pulses (76.5%), and whole grains (65%), whereas fewer consumed fruits (43.3%) and nuts or seeds (9.8%). However, a significant proportion consumed sweet foods (45.5%) and deep‐fried foods (19.3%), with relatively low consumption of salty snacks, instant noodles, fast foods, and soft drinks, all of which are considered risk factors for NCDs.

The majority of the participants (57.3%) reported having debt. Financial support was the most expected assistance to improve living standards (52.5%), followed by cattle (14.8%) and land (9.5%). Plans for utilizing financial assistance were predominantly focused on business (40.9%) and agro farming (28.9%). A large proportion of participants (80.5%) reported no need for training to increase income; however, among those who required training, agro farming (42.3%) was the most commonly cited, followed by business‐related skills (21.8%) and driving (21.8%) (Table 3).

**TABLE 3:** Distribution of debt status, expected support, and plans for improving living standards among study participants.

Characteristics	Percent
**Have any debt**	
Yes	57.3
No	42.7
**Kind of support expected to increase standard of living**	
Financial	52.5
Cattle	14.8
Land	9.5
Auto rickshaw	7.8
Nothing	14
Others	1.5
**Plan for utilizing financial assistance**	
Business	40.9
Agro farm	28.9
Buying an auto rickshaw/CNG	15.7
Repayment of loan and house repairing	7.7
Family expenses	6.8
**Training needed to increase income**	
Yes	19.5
No	80.5
**Types of training required**	
Agro farming	42.3
Business related	21.8
Driving	21.8
Any other work	14.1

As shown in Table 4, both education level and household monthly income were significantly associated with FI. Participants who had attained secondary education had significantly lower odds of experiencing FI (AOR = 0.56, 95% CI: 0.31–0.79, *p* = 0.042) compared to those with no formal education. Similarly, individuals from households with a monthly income exceeding BDT 25,000 were substantially less likely to experience FI (AOR = 0.20, 95% CI: 0.08–0.49, *p* < 0.001) compared to those with an income of BDT 16,000 or less.

**TABLE 4:** Factors associated with food insecurity among the study participants.

Variable	Food insecurity *n* (%)	COR (95% CI)	*p* value	AOR (95% CI)	*p* value
**Education level**					
No formal education	152 (75.2)	1		1	
Primary	91 (79.8)	1.30 (0.74, 2.27)	0.355	1.20 (0.67, 2.14)	0.524
Secondary	56 (66.7)	0.65 (0.37, 1.14)	0.139	0.56 (0.31, 0.79)	0.042
**Earning members**					
Single	199 (75.1)	1		1	
Two or more	100 (74.1)	0.94 (0.58, 1.52)	0.824	1.80 (0.97, 3.32)	0.059
**Working hour**					
≤8	112 (76.7)	1		1	
>8	187 (73.6)	0.84 (0.52, 1.36)	0.494	0.81 (0.49, 1.33)	0.414
**Household monthly income (BDT)**					
Up to 16,000	85 (81.0)	1		1	
16,001–20,000	86 (78.9)	0.88 (0.45, 1.71)	0.708	0.70 (0.35, 1.41)	0.321
20,001–25,000	78 (74.3)	0.68 (0.35, 1.30)	0.248	0.43 (0.20, 0.90)	0.027
>25,000	50 (61.7)	0.38 (0.19, 0.73)	0.004	0.20 (0.08, 0.49)	<0.001
**Household monthly food expenditure (BDT)**					
<5000	34 (91.9)	1		1	
5000–8000	136 (81)	1.07 (0.48, 2.40)	0.857	1.14 (0.50, 2.63)	0.743
8001–10,000	101 (84.9)	1.46 (0.62, 3.43)	0.379	1.88 (0.76, 4.68)	0.171
>10,000	56 (73.7)	0.80 (0.33, 1.91)	0.621	1.35 (0.50, 3.66)	0.546

Abbreviations: AOR, adjusted odds ratios; BDT, Bangladeshi taka; CI, confidence interval; COR, crude odds ratios.

Table 5 demonstrates that education level, number of earning members in the household, and monthly household income were significantly associated with food group diversity. Individuals with primary (AOR = 2.71; 95% CI: 1.63–4.51; *p* < 0.001) and secondary education (AOR = 2.74; 95% CI: 1.53–4.91; *p* = 0.001) had significantly higher odds of achieving adequate food group diversity compared to those with no formal education. Households with two or more earning members were also more likely to consume a diverse diet than those with only one earner (AOR = 2.30; 95% CI: 1.32–4.01; *p* = 0.003). Additionally, higher household income was negatively associated with dietary diversity. Specifically, individuals from households income BDT 16,000–20,000 (AOR = 0.34; 95% CI: 0.19–0.63; *p* < 0.001) and BDT 20,001–25,000 (AOR = 0.32; 95% CI: 0.17–0.60; *p* < 0.001) were less likely to report adequate food group diversity compared to those with an income of BDT 16,000 or less.

**TABLE 5:** Factors associated with food group diversity of the study participants.

Variable	Food group diversity (≥5 food groups) *n* (%)	COR (95% CI)	*p* value	AOR (95% CI)	*p* value
**Age (year)**					
<30	38 (56.7)	1		1	
30–45	98 (48.0)	0.70 (0.40, 1.23)	0.219	1.04 (0.56, 1.91)	0.891
≥46	69 (53.5)	0.87 (0.48, 1.59)	0.667	1.31 (0.67, 2.55)	0.430
**Education level**					
No formal education	82 (40.6)	1		1	
Primary	69 (60.5)	2.24 (1.40, 3.58)	0.001	2.71 (1.63, 4.51)	<0.001
Secondary	54 (64.3)	2.63 (1.55, 4.46)	<0.001	2.74 (1.53, 4.91)	0.001
**Nutritional knowledge level**					
Poor	68 (50.4)	1		1	
Average	85 (47.2)	0.88 (0.56, 1.37)	0.58	0.85 (0.52, 1.37)	0.512
Good	52 (61.2)	1.55 (0.89, 2.69)	0.118	1.60 (0.88, 2.92)	0.120
**Earning members**					
Single	121 (45.7)	1		1	
Two or more	84 (62.2)	1.96 (1.28, 2.99)	0.002	2.30 (1.32, 4.01)	0.003
**Household monthly income (BDT)**					
Up to 16,000	64 (61.0)	1		1	
16,001–20,000	47 (43.1)	0.48 (0.28, 0.83)	0.009	0.34 (0.19, 0.63)	<0.001
20,001–25,000	44 (41.9)	0.46 (0.26, 0.80)	0.006	0.32 (0.17, 0.60)	<0.001
>25,000	50 (61.7)	1.03 (0.57, 1.87)	0.914	0.57 (0.26, 1.22)	0.150
**Food insecurity**					
Food secure	42 (57.5)	1		1	
Food insecure	163 (49.8)	0.93 (0.59, 1.47)	0.776	0.88 (0.53, 1.45)	0.623

Abbreviations: AOR, adjusted odds ratios; BDT, Bangladeshi taka; CI, confidence interval; COR, crude odds ratios.

## 4 Discussion

This study explored the concurrent issues of FI, diet quality, and nutritional status among rickshaw pullers in Dhaka city. The findings indicate that approximately 60% of participants experienced MFI to SFI, with 6.8% classified as severely food insecure. Moreover, a significant proportion (57.3%) reported being in debt, and more than half (52.5%) anticipated financial support to improve their living standards. Notably, higher educational attainment and higher household income were significantly associated with lower odds of experiencing FI. Additionally, nearly half (48.8%) of the participants consumed a diet with fewer than five food groups, signifying low dietary diversity. Higher education levels and a greater number of earning members were significantly associated with better dietary diversity. In terms of nutritional status, 8.8% of participants were underweight, whereas 10.4% were overweight, reflecting a dual burden of malnutrition within this population.

This study found a high prevalence of FI among the participants, with approximately 60% experiencing MFI to SFI, including 6.8% classified as severely food insecure. The high prevalence of FI observed in this study is consistent with previous research, which found that over 67% of low‐income households in Bangladesh face mild to MFI [25]. The elevated levels of FI among rickshaw pullers may be attributed to their socioeconomic vulnerability, as they are predominantly low‐income earners residing in slum areas [15]. This precarious economic status likely exacerbates their risk of FI, contributing to a higher prevalence compared to the national average, where 20% of the population experiences acute FI [26].

Furthermore, the high levels of indebtedness and financial instability among participants further underscore their economic vulnerability. This study found that 57.3% of participants reported being in debt, and more than half (52.5%) anticipated financial support to improve their living standards, aligning with findings of a previous study that reported that 70% of rickshaw pullers in Dhaka live in debt, whereas 96% experience financial deficiency [27]. Such financial constraints not only limit access to adequate food but also contribute to poor overall living conditions, further reinforcing cycles of poverty and malnutrition.

Education emerged as a significant protective factor against FI. Participants with secondary education had lower odds of experiencing FI compared to those with no formal education. Higher education can improve financial literacy and decision‐making skills, enable individuals to manage resources effectively, budget for food expenses, and access social safety nets when needed. Additionally, educated individuals are more likely to be aware of nutrition, food safety, and available assistance programs [28], which can further reduce their vulnerability to FI.

Consistent with previous research, our study found that higher household monthly income is significantly associated with reduced levels of FI [29, 30]. Income plays a pivotal role in determining a household's ability to access sufficient and nutritionally diverse food. In low‐ and middle‐income countries such as Bangladesh, it serves as a key driver of both food availability and dietary quality. Households with higher income levels are typically more resilient to food price fluctuations, can invest in food storage and transportation, and have greater access to a variety of food sources [31]. These advantages collectively contribute to a lower risk of FI. In contrast, economically disadvantaged households often face financial constraints that force them to compromise on both the quantity and quality of food consumed, increasing their susceptibility to inadequate nutrient intake and malnutrition.

Regarding diet quality, this study found that approximately half of the participants consumed a diet comprising fewer than five food groups, indicating a potential risk of micronutrient deficiencies. Although nearly all participants (99%) consumed grains daily, the intake of fruits, vegetables, animal‐source foods, and pulses was notably low. These findings are in line with a previous research on rickshaw pullers in Dhaka, which reported that cereals serve as the primary sources of energy, protein, and micronutrients, yet overall diets remain deficient in energy and essential nutrients [32]. The low dietary diversity among rickshaw pullers may be attributed to economic constraints limiting access to nutrient‐rich foods, long working hours restricting meal variety, low nutritional awareness, limited availability of diverse foods in their living and working environments, and cultural dietary habits favoring staple foods.

Participants with primary or secondary education exhibited better dietary diversity than individuals with no formal education. This result is consistent with findings from several studies indicating that higher education is a significant determinant of improved dietary habits [33, 34, 35, 36]. Higher levels of education can lead to an increased awareness of the importance of balanced nutrition, better access to information on healthy eating, and improved decision‐making skills related to food choices. Therefore, expanding educational opportunities could be a useful policy strategy to promote healthy eating behavior.

Additionally, households with multiple earning members demonstrated significantly higher dietary diversity than those with a single earner. Income plays a crucial role in determining dietary diversity, as higher household earnings enable greater access to diverse and nutrient‐rich foods [33, 37, 38]. Households with multiple income sources can allocate more financial resources toward purchasing a variety of foods, such as fruits, vegetables, and protein‐rich items, which are often less accessible to low‐income populations. In contrast, single‐earner households may face budget constraints that limit their ability to afford a nutritionally diverse diet.

Interestingly, this study found that higher household income was negatively associated with dietary diversity, which contrasts with findings from previous research [39]. One possible explanation for this counterintuitive result is that individuals in low‐income groups—such as migrant workers or daily wage earners—often prioritize financial obligations over dietary choices. A significant portion of their earnings may be allocated to repaying loans or sending money to family members, leaving limited resources for purchasing a variety of foods. As a result, they may rely on simple, repetitive meals that do not reflect a diverse diet, despite having relatively higher income compared to others in their peer group.

The study also revealed a coexistence of both undernutrition and overnutrition among the participants, with 8.8% classified as underweight and 10.4% as overweight. These findings are nearly consistent with a previous study that examined rickshaw pullers in Dhaka city, where 11.5% were underweight and 23.1% were overweight or obese [10]. The presence of both forms of malnutrition within this population underscores the growing dual burden of malnutrition, which is increasingly evident in urban settings. Socioeconomic disparities, poor dietary patterns, inconsistent food availability, lack of nutritional awareness, and cultural influences may contribute to this phenomenon [40, 41].

### 4.1 Public Health Implications

Given the context of high FI, poor diet quality, and moderate levels of malnutrition, this study underscores the urgent need for multi‐sectoral action to address nutritional disparities among rickshaw pullers in Dhaka. Community‐based nutrition education and workplace initiatives such as providing affordable, healthy meals at rickshaw stands can promote better dietary practices. Financial support mechanisms, like microcredit or income‐generating activities, may also help reduce FI by enhancing economic stability.

From a policy perspective, expanding social safety nets and food assistance programs for low‐income urban workers is essential. Improving access to education and financial literacy could provide long‐term benefits. To address the double burden of malnutrition, regular health and nutrition assessments should be integrated into existing health services. Sustainable improvements require a comprehensive approach that blends immediate interventions with long‐term socioeconomic development to ensure equity and resilience among this vulnerable group.

### 4.2 Strengths and Limitations

This study has several strengths, including being the first to assess FI among rickshaw pullers using the validated FIES, ensuring reliable measurement. It covers a broad area of Dhaka city, enhancing the generalizability of findings, and employs the validated DQQ to evaluate dietary quality. It also identified the types of assistance participants expect, offering practical insights for policymaking. However, limitations include its cross‐sectional design, which restricts causal inference, and the use of convenience sampling, which may introduce selection bias and limit representativeness. Additionally, lifestyle factors such as alcohol and tobacco use were not assessed due to cultural sensitivities in Bangladesh, which may limit insight into their impact on income and expenditure. Despite these constraints, the study provides critical evidence to inform policies and interventions aimed at improving the well‐being of this vital workforce in Bangladesh.

## 5 Conclusion

This study indicates that there is a significant FI and financial hardship among the participants, with the majority experiencing MFI to SFI and a considerable proportion relying on debt to meet their basic needs. Dietary diversity was notably low, with nearly half of the participants consuming fewer than five food groups. The coexistence of undernutrition and overweight within the population reflects the complex nutritional challenges they face. Addressing these issues requires targeted interventions, including food assistance programs, financial support, and skill development initiatives. Policies should focus on expanding income opportunities, promoting workplace nutrition programs, and enhancing access to affordable, diverse diets.

## Author Contributions

All authors made a significant contribution to this work, including involvement in the study's conception and design, data collection, analysis, and interpretation. Each contributed to drafting and revising the manuscript, reviewed it critically for important intellectual content, and approved the final version for publication.

## Ethics Statement

Ethical approval for this study was obtained from the Institutional Review Board of the Faculty of Biological Sciences, University of Dhaka (Ref. No. 299/Biol. Scs.).

## Consent

Participation was voluntary, and the nature and objective of the study were thoroughly explained to all participants, and informed consent was obtained from each individual prior to their involvement in the study.

## Conflicts of Interest

The authors declare no conflicts of interest.

## Supporting information




**Supporting Figure 1**: puh270149‐sup‐0001‐Figures.png

**S. Table 1**: Assumptions check for regression model 1 (Factors associated with food insecurity). **S. Table 2**: Assumptions check for regression model 2 (Factors associated with dietary diversity).

## Data Availability

Data and materials are available upon reasonable request to the corresponding author.
