# Supplementary figures and images for: Exploring Food Insecurity and Nutritional Challenges Among Rickshaw Pullers in Dhaka City, Bangladesh
Source: Public Health Chall. 2025 Oct 22;4(4):e70149. doi: 10.1002/puh2.70149 (PMC12542296; doi:10.1002/puh2.70149)

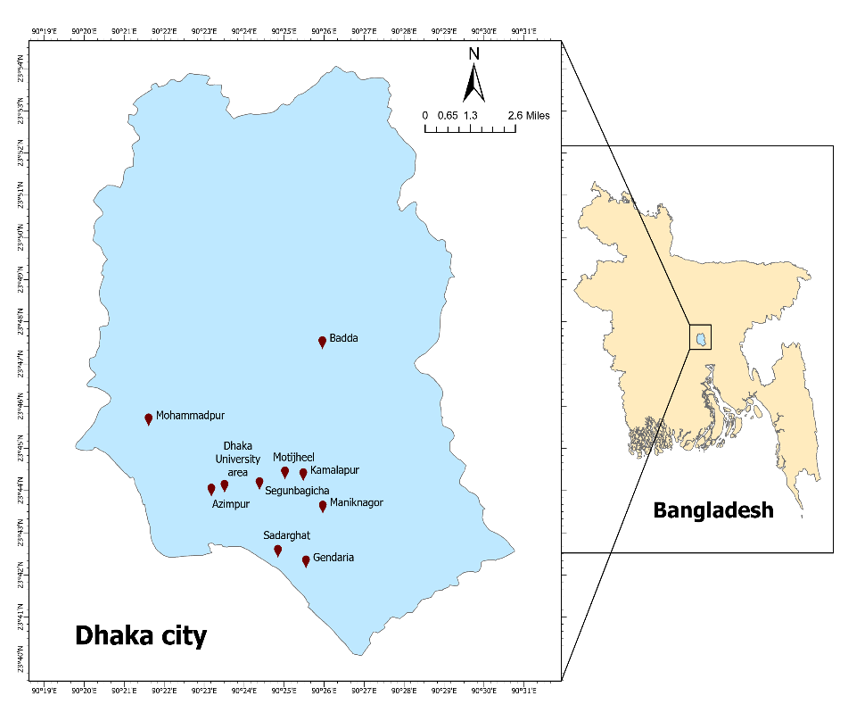

Supplement: Supplementary file 1 — Supporting Figure 1: puh270149‐sup‐0001‐Figures.png [file PUH2-4-e70149-s001.png]
