# Supplementary material for: Exploring Food Insecurity and Nutritional Challenges Among Rickshaw Pullers in Dhaka City, Bangladesh
Source: Public Health Chall. 2025 Oct 22;4(4):e70149. doi: 10.1002/puh2.70149 (PMC12542296; doi:10.1002/puh2.70149)
Supplement: Supplementary file 2 — S. Table 1: Assumptions check for regression model 1 (Factors associated with food insecurity). S. Table 2: Assumptions check for regression model 2 (Factors associated with dietary diversity). [file PUH2-4-e70149-s002.pdf]

S. Table 1. Assumptions check for regression model 1 (Factors associated with food insecurity)

Factors	Collinearity statistics		Hosmer and Lemeshow test	Nagelkerke R square	Omnibus test
	Tolerance	VIF			
Education level	0.97	1.02	0.563	0.810	0.012
Earning member	0.67	1.47			
Working hour	0.99	1.00			
Monthly income	0.58	1.71			
Monthly food expenditure	0.73	1.35			

S. Table 2. Assumptions check for regression model 2 (Factors associated with dietary diversity)

Factors	Collinearity statistics		Hosmer and Lemeshow test	Nagelkerke R Square	Omnibus test
	Tolerance	VIF			
Age	0.88	1.13	0.113	0.164	< 0.001
Education level	0.89	1.12			
Earning member	0.66	1.50			
Nutritional knowledge level	0.96	1.03			
Monthly income	0.66	1.51			
Food insecurity	0.94	1.05			

For Review Only
